# Introducing Structural Reliance: A New Method to Assess Structure–Function Coupling in the Brain

**DOI:** 10.1002/hbm.70499

**Published:** 2026-03-26

**Authors:** Derek Madden, Paul J. Laurienti, Heather M. Shappell, Mohsen Bahrami

**Affiliations:** ^1^ Virginia Tech‐Wake Forest University School of Biomedical Engineering and Sciences, Wake Forest University School of Medicine Winston‐Salem North Carolina USA; ^2^ Department of Radiology Wake Forest University School of Medicine Winston‐Salem North Carolina USA; ^3^ Department of Biostatistics and Data Science Wake Forest University School of Medicine Winston‐Salem North Carolina USA

**Keywords:** fMRI, functional connectivity, multivariate autoregressive model, structural connectivity, structure–function coupling

## Abstract

The relationship between the structural connectome and functional activity in the brain is highly complex, and understanding of the connection between the two is limited. Previous work has shown a marginal reliance of functional brain activity on underlying structural connections, indicating significant flexibility of neural communication. Here, we introduce a new method to quantify structure–function coupling and compare it with a standard coupling technique by evaluating the structure–function relationship across numerous fMRI task paradigms. Through this comparison, we investigate how structure–function relationships change during different cognitive demands and we evaluate how they relate to behavior. The new method introduced here, structural reliance, exhibits different structure–function correspondence patterns throughout the brain, and it generally outperforms the standard coupling measure in coupling‐based behavioral measure predictions.

## Key Points

- The relationship between brain structure and function is complex and imperfectly described with current coupling methods.
- Structural reliance provides an alternative measure to quantify structure–function coupling in the brain that embraces dynamic functional patterns.
- Structural reliance generally outperformed standard correlation coupling in predicting various subject cognitive measures and demographic variables.

## Graphical abstract

Structural reliance is a new structure–function coupling technique for use in brain imaging research that utilizes brain activity in neighboring regions to predict concurrent seed activity based on the strength of structural connections. This new technique generally outperformed standard coupling in behavioral predictions.

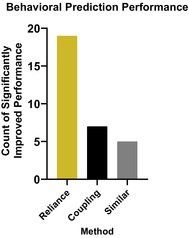

## 1 Introduction

Many believe that the structural anatomy of the brain dictates its functional patterns. However, the flexibility of brain function and imperfect correspondence between structure and function indicate a complex relationship that has proven difficult to capture. In addition to the difficulty of quantifying structural connections in the brain, one must also differentiate between inhibitory versus excitatory, active versus inactive, and strong versus weak connections (Brofiga et al. 2022). Previous efforts to research this relationship have explored how diffusion‐weighted magnetic resonance imaging (DWI) measured structural connectivity (SC) relates to functional connectivity (FC) measured by blood oxygen level‐dependent (BOLD) functional magnetic resonance imaging (fMRI) (Baum et al. 2020; Fotiadis et al. 2024; Gu et al. 2021; Kong et al. 2021; Liu et al. 2022; Popp et al. 2024; Wang et al. 2023). Primarily, the correlation between a given region's SC and FC profiles has been evaluated to measure the correspondence between a region's functional communication and its structural connections to neighboring regions. Given a direct relationship, regions exhibiting a stronger structural connection would be assumed to exhibit a stronger functional connection as well. This measure, deemed SC‐FC coupling, has been related to a variety of behavioral functions, from general cognitive ability to neurological disease (Kong et al. 2021; Popp et al. 2024; Wang et al. 2023).

Studies evaluating SC‐FC coupling indicate that while function is not directly adherent to structure, functional performance can be related to the level of reliance that function exhibits on structure. The direction of this relationship, however, may vary depending on the behavioral measure being evaluated. As cognitive flexibility, or the capacity to adjust behavior based on changing surroundings, is highly associated with overall cognitive function and performance on individual tasks, it is imperative that healthy brain function exhibits flexibility in its adherence to structural connections rather than a static or complete adherence to the structural connectome (Dajani and Uddin 2015). Similarly, studies have shown that task performance increases for individuals whose functional networks differ greatly between rest and alternative tasks (Saggar et al. 2018), showing that increased functional flexibility is associated with improved cognitive performance. Thus, cognitive function is related to the brain's ability to function according to many different FC patterns that may differ from structural anatomy. Meanwhile, when tasks evoke neural patterns that are well‐defined by the structural connections, functional communication through strong structural connections may be more effective, resulting in a positive relationship between coupling and performance.

While recent coupling research has aided in progressing knowledge, understanding of the connection between structure, function, and behavior remains incomplete. Here, we suggest that current methods for examining SC‐FC coupling overlook a critical aspect of the structure–function relationship, largely due to how FC is constructed and applied in SC‐FC analyses. For one, while FC is a strong, simple measure to evaluate the similarity between two regions' functional time series, it serves as a static estimation of regional intercommunication spanning an entire time span. Communication in the brain is constantly evolving based on cognitive demands, so a more dynamic evaluation of functional communication may be better suited to understand how functional patterns adhere to the structural connectome at different times (Shappell et al. 2021). Additionally, FC does not necessarily indicate communication between two regions (Mohanty et al. 2020). Often, regions may share an input that largely dictates their functions, resulting in apparent functional connection despite no structural connection or intercommunication. More broadly, neurons communicate within a large network, where they receive and send a multitude of signals that results in a complex combination of influence exerted upon each region (Caire and Reddy 2025; Pessoa 2023; Shapson‐Coe et al. 2024). Given the multitude of connections and high neuronal density within a voxel or parcellated atlas region, extensive communication between two regions may not result in similar functional activity, while other regions may exhibit very similar FC without any direct intercommunication. The diverse influences from numerous regions transmitting distinct signals likely result in considerable variability in how communicating regions respond. Alternative coupling methods have incorporated network measures like shortest path length to account for mutual neighbors by quantifying the strength of all possible avenues of information travel between regions rather than strictly direct communication (Fotiadis et al. 2024; Popp et al. 2024; Rubinov and Sporns 2010; Seguin et al. 2020; Suarez et al. 2020; Wang et al. 2025; Zamani Esfahlani et al. 2022). Still, these approaches also inflate structural connection values between regions that are not functionally homogenous but are connected by a single internetwork pathway, resulting in only slight improvements in the variance in function explained by structure. Regardless of alternative representations of the structural connectome, FC can be prone to misrepresenting communication between regions due to its lack of inclusion of all regions during calculation (Mohanty et al. 2020).

Various alternatives to FC have been utilized in research to better consider the activity of all regions in quantifying communication. Partial correlation, for instance, considers similarity of activity between regions while controlling for the activity of remaining brain regions (Ryali et al. 2012; Wang et al. 2016). However, partial correlation is most effective at eliminating false positives (no direct communication but high FC) connections by controlling for neighbors that may dictate function for two unconnected regions. Equally important is a metric that eliminates false negatives by considering how functionally heterogeneous, communicating regions may exhibit different functional activity due to an overwhelming number of connections with functionally homogeneous regions, perhaps within a subnetwork. One technique that limits both false positives and false negatives (direct communication but low FC) regional communication values is the multivariate autoregressive (MAR) model, which reconstructs a region's BOLD time series based on its prior activity and the activity of other brain regions, thereby considering dynamic, multivariate influences across the brain (Chiang et al. 2017; Crimi et al. 2021; Harrison et al. 2003; Li et al. 2014; Wee et al. 2013). Moreover, communication between regions occurring via a mutual neighbor will be reflected in the neighbor's time series, helping to address this limitation as well. When utilized alongside Granger causality, the model allows the user to determine the influence exerted by each region on another, resulting in a connectivity measure referred to as effective connectivity (EC) (Harrison et al. 2003; Li et al. 2014). Building on this research, more recent studies have leveraged SC to constrain EC, setting EC values between regions lacking structural connections to zero to better estimate communication (Chiang et al. 2017; Crimi et al. 2021). While such work has shown the capacity to use these measures in tandem, the impact of this approach and its potential to enhance our understanding of SC‐FC coupling remains largely unexplored.

Here, we present a novel method for reconstructing functional activity by implementing SC within a modified MAR framework and examining its implications for redefining SC‐FC coupling. We show that this method can serve as both an alternative and a complement to existing SC‐FC coupling measures. After reconstructing regional time series using an SC‐weighted sum of other regions' time series within an MAR framework, we determine the correlation between each region's reconstructed (predicted) time series and its true time series. This correlation coefficient, referred to hereafter as *structural reliance*, estimates how regional activity adheres to underlying structural pathways. In other words, this metric quantifies the extent to which functional activity of a region *relies* on the sum of its structural connections, where high structural reliance values indicate regions that appear to communicate more directly in accordance with SC values.

To demonstrate the efficacy of this method in quantifying the structure–function relationship and its connection to behavior, we use the Human Connectome Project (HCP) data (Van Essen et al. 2013) to compare traditional SC‐FC coupling values to our structural reliance values across different regions and evaluate the relationship between coupling/reliance and function by using these values to predict performance in fMRI tasks and behavioral measurements. Specifically, we use structural reliance and standard SC‐FC coupling data to predict subject fMRI task accuracy, overall cognition, and various demographic measures. fMRI task accuracy provides an outcome variable directly related to the cognitive demands evoked during imaging, ensuring relevant functional patterns in the brain. Overall cognition serves as a behavioral measure that has been extensively studied in structure–function coupling and is highly related to cognitive load exerted during fMRI tasks (Liu et al. 2024; Popp et al. 2024; Sun et al. 2024; Wang et al. 2023; Wu et al. 2023). We also investigated age, body mass index (BMI), and frequency of heavy drinking in the past 12 months as demographic variables. Age is well‐studied with respect to structure and function, both independently and together through coupling (Baum et al. 2020; Liu et al. 2024; Sun et al. 2025, 2024; Wang et al. 2025). Frequency of heavy alcohol consumption and BMI, on the other hand, served as investigative variables that, despite multiple investigations into their relationship with brain structure and function independently, have limited research on their connection to the structure–function relationship (Chen et al. 2018; Kim et al. 2024; Kirse et al. 2023; Laurienti et al. 2023; Maleki et al. 2022; Namgung et al. 2024; Park et al. 2016; Ronan et al. 2020; Topiwala et al. 2022; Yip et al. 2023; Yoon et al. 2020; Zhang et al. 2022). Together, these measures provided a wide range of variables to investigate structural reliance and coupling and how they relate to behavior.

## 2 Materials and Methods

### 2.1 Participants and Data Acquisition

Analysis was conducted using data from the HCP Young Adult S1200 dataset, which includes 1200 individuals aged 22–37 (Van Essen et al. 2013). Of those, 1113 including 606 females have complete MRI, cognitive testing, and detailed demographic data. A subset of 1034 individuals (559 females, 475 males, average age of 28.8 years) with complete diffusion tensor imaging (DTI) was utilized in this study. We used minimally preprocessed DTI and fMRI data provided by the HCP study (Glasser et al. 2013). Within the subset of 1034 participants, few were missing any resting‐state fMRI, task‐based fMRI, or behavioral data. Given that different analyses relied on different tasks or behavioral analyses, participants were included in each analysis based on available data for that task/behavior to maximize sample size. Participant counts were very similar across the different fMRI paradigms, ranging from a minimum of 1005–1026. See participant counts for each task in the supplemental file (Table S1). All data were collected on 3T Siemens MRI scanners located at Washington University or the University of Minnesota. The MRI data were obtained with a Siemens Skyra 3T scanner using a gradient‐echo EPI sequence and 32‐channel head coil. Functional data (BOLD‐weighted) were obtained from the minimally preprocessed fMRI data acquired using the following parameters: TR = 720 ms, TE = 33.1 ms, voxel size = 2 × 2 × 2 mm, flip angle = 52°, and multiband acceleration factor = 8 (Glasser et al. 2013). DTI were obtained from the minimally preprocessed DWI data with the following parameters: TR = 5520 ms, TE = 89.5 ms, voxel size = 1.25 × 1.25 × 1.25 mm, multiband acceleration factor = 3, *b* = 1000, 2000, 3000 s/mm^2^, and 90 directions/shell (Glasser et al. 2013).

### 2.2 Structural and Functional Network Construction

We used the Multimodal Surface Mapping (MSMAll)‐registered version of the minimally preprocessed fMRI data provided by the HCP study, which registers denoised BOLD time‐series across individuals. Specifically, this approach uses surface‐based multimodal inter‐participant registration to enhance interparticipant alignment by incorporating both anatomical and functional information (Robinson et al. 2014). To further denoise the data, participant motion parameters (along with their first‐order derivatives) and global, ventricular, and white matter signals were also regressed from the data (Power et al. 2018). By regressing global signal, we ensured that reliance and coupling values more accurately relate structural and functional connections rather than inflating reliance/coupling coefficients based on correlation to global signal. Resting‐state BOLD signals were filtered using a standard band pass filter (0.009–0.08 Hz) to reduce physiological noise. More details about the BOLD signals, including the length of time series and other specifications for the resting‐state and task data are provided in the supplemental file (Table S1). The brain was then parcellated into 360 regions using the MMP atlas (Glasser et al. 2016), and BOLD signals were averaged within each region to generate regional time series. A functional network for each participant was constructed from the MMP time series by computing the Pearson correlation between all pairs regions, resulting in a 360 × 360 correlation matrix, and the Fisher‐Z transformation was applied to normalize the values (Madden et al. 2025).

The minimally preprocessed diffusion data includes BedpostX outputs that model fiber orientation with FSL's multishell spherical deconvolution toolbox (Jbabdi et al. 2012). Using BedpostX outputs, we performed probabilistic tractography via FSL's probtrackX (Behrens et al. 2007) to generate a dense structural connectome for each participant. Specifically, using 91,282 Grayordinates (comprising 32,492 white matter/gray matter boundary surfaces per hemisphere and 27,298 subcortical voxels), we performed whole‐brain probabilistic tractography by seeding from each Grayordinate and tracking to all others. The resulting 91,282 × 91,282 matrix shows the number of streamlines connecting each pair of Grayordinates. This dense whole‐brain connectome can subsequently be parcellated according to any desired atlas to derive the region‐wise connectivity. We used the MMP atlas to parcellate into 360 regions and derive a 360 × 360 structural network for each individual. For each pair of regions, the total number of streamlines was computed by summing all streamlines originating from one region and terminating in the other. This yielded a directed, weighted atlas‐based SC matrix. Dense connectomes were generated using a high‐performance computing cluster. For each individual, 100 instances of the ProbtrackX command were distributed across the cluster using different random seeds (ensuring different stochastic results), with each seed having 50 samples. The results were then combined using FSL to generate a dense connectome (with 5000 curves per seed). Please see the supplement for additional detail about the tractography and generating dense connectomes.

### 2.3 Coupling

The SC profile of a region (${\mathrm{SC}}_{i}$) was defined as a vector containing its SC values to all other parcellated regions; similarly, its FC profile consisted of the FC values to all other regions (${\mathrm{FC}}_{i}$). SC‐FC coupling for each region was then calculated as Pearson's correlation between these two profiles (i.e., ${\mathrm{cp}}_{i}=\text{corr}\left({\mathrm{SC}}_{i},{\mathrm{FC}}_{i}\right)$). We also considered using Spearman's correlation, but investigative analysis revealed that Pearson's correlation outperformed Spearman's in the framework of this work (see Supplement for details). Higher coupling values for a given region indicate that the strength of SC patterns to neighbors were highly associated with the strength of FC patterns.

### 2.4 Reliance

This work introduces a technique which builds on previous MAR‐based fMRI research (Chiang et al. 2017; Crimi et al. 2021; Harrison et al. 2003; Li et al. 2014; Wee et al. 2013) to yield a single correlation coefficient for each region that measures the adherence of functional activity to the structural connections, akin to the basic coupling value described above. The measure aims to quantify how the behavior arising from the functional activity of a region is *reliant* on its structural connectome; thus, we refer to the technique as *structural reliance*.

(1) $$\hat{i_{\mathrm{Rel}}}\left(t+1\right)=\hat{i_{\mathrm{Rel}}}\left(t\right)+\mathrm{TR}\times \sum_{j=1}^{N}\frac{\mathrm{dj}\left(t\right)}{\mathrm{dt}}\times \left(\frac{{\mathrm{sc}}_{\mathrm{ji}}}{\sum_{k=1}^{N}{\mathrm{sc}}_{\mathrm{ki}}}\right).$$

**FIGURE 1:**
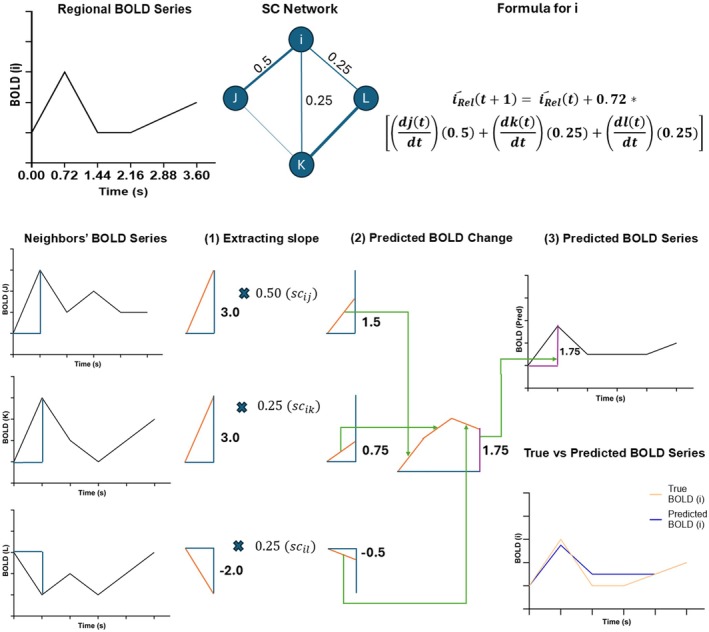
Example generation of the predicted BOLD time series for a given region, *i*, using structural reliance workflow. (1) At each TR, the first derivative of the BOLD series for all neighboring regions is multiplied by the structural connectivity value between the neighboring region and the seed region. (2) The sum of weighted derivatives is calculated as the predicted change in BOLD signal of the seed region between the current and next TR. (3) By iterating across each TR, a full predicted time series is generated, and the correlation between this predicted time series and the true time series is then obtained as the structural reliance value.

To quantify structural reliance of region, we first use Equation (1) to generate a predicted BOLD time series for the region based on the structural connections to and function of its neighbors. Rather than standard MAR, which displaces each time series by some number of TRs to predict future BOLD activity, we predict a region's concurrent activity by calculating a weighted sum of the first derivatives of the BOLD time series ($\frac{\mathrm{dj}\left(t\right)}{\mathrm{dt}}$) from all remaining N regions, where the derivative is calculated as the slope of the BOLD series between time points t and t+1. Weights are assigned as the SC values (${\mathrm{sc}}_{\mathrm{ji}}$) from each region to the seed region, normalized by the sum of all SC values to the seed ($\sum_{k=1}^{N}{\mathrm{sc}}_{\mathrm{ki}}$). The derivative reflects changes in activity, so at each time point ***t***, the weighted sum represents how changes in the activity of other brain regions, exerted through structural connections, predict the rate of change in BOLD signal of the seed region at that timepoint. Starting from the true time series value at time 0, the weighted sum of the derivatives from the neighbors of node i is multiplied by the ***TR*** to estimate the change in BOLD signal between the current and next time point. This change estimate is added to the current BOLD value of node i to estimate the BOLD signal value at time t+1. This process is iteratively repeated for the remainder of the time series, resulting in a complete predicted BOLD time series ($\hat{i_{\mathrm{Rel}}}$). A Pearson correlation is then computed between the reconstructed/predicted time series and the region's true BOLD time series, yielding a single correlation coefficient for the region (i.e., ${\mathrm{rel}}_{i}=\text{corr}\left(\hat{i_{\mathrm{Rel}}},i\right)$). An example of this workflow is demonstrated below in Figure 1.

### 2.5 Comparison of Magnitude Patterns

The first comparison between coupling and structural reliance involved evaluating the magnitude patterns of reliance and coupling values across the brain. Reliance and coupling may both reflect spatial correspondence in structural and FC patterns, but structural reliance employs BOLD dynamics to produce a time‐resolved metric of structure–function association that embraces changes in regional communication while coupling measures the static correlation between connectivity profiles. The dynamic nature of reliance may offer complementary insights into how a region's structural architecture supports its regional function over time, but its use of the functional activity of other regions to predict activity of seed region prevents direct comparison of magnitudes of each measure. However, comparing the relative magnitudes of each measure is useful in demonstrating the novelty of the reliance measure and the distinct patterns of structure–function correspondence in the brain for both methods. The magnitude of regional values relative to other regions is critical to understanding which regions are more reliant on their structural connections during functional communication. For the resting‐state data, we computed both coupling and structural reliance values for all 360 parcellated regions in each participant with acceptable resting‐state data. Coupling and structural reliance values for each region were separately averaged across participants to estimate a typical value for each region. With group average correlation values for each region, we ranked regions by the average magnitude of coefficients to discern which regions differed between reliance and coupling in their relative magnitude.

The magnitude patterns of coupling and reliance coefficients were also compared across different fMRI task paradigms. To explore the flexibility in the association between structure and function, we calculated average whole‐brain coupling and reliance coefficients for each participant within each of the eight fMRI paradigms of the HCP study. The mean standard deviation between regional coefficients was also calculated within participants to examine how structure–function correspondence varies between different regions. Finally, we calculated the standard deviation of coupling and reliance values between tasks for both participant brain‐average coefficients and individual regional coefficients within participants, enabling investigation into how structure–function correspondence differs under varying cognitive demands.

### 2.6 Predicting Participant Measures Using Reliance Values

SC‐FC coupling and structural reliance were compared in their capacity to predict a variety of participant measures: fMRI task performance, overall cognition, and demographic measures. Of the seven fMRI tasks conducted in the HCP, five returned quantifiable performance metrics: Emotional processing, language, relational processing, social cognition, and working memory (Barch et al. 2013; Van Essen et al. 2013). Short descriptions of each task and performance metrics are provided in Table 1. For the five performance‐based tasks, overall accuracy was selected as the metric for predictions, where applicable. For the social cognition task, the sum of correct response percentages was subtracted by the sum of incorrect response percentages, and unsure response percentages were not included in the sum. Participant's overall cognition was estimated using their age‐adjusted NIH Toolbox Cognition Total Composite Score (Akshoomoff et al. 2013). Combining performance on both fluid and crystallized cognitive measures, the composite score represents an individual's general cognitive ability based on performance across a multitude of different cognitive demands. Finally, we chose to compare coupling and structural reliance measures in their ability to predict performance in three demographic measures not directly related to tasks: Age, BMI, and frequency of heavy alcohol consumption. Age was measured in years, BMI measured as a participant's weight in kilograms divided by the square of the participant's height in meters, and frequency of heavy alcohol consumption measured as reported number of times being drunk in the past 12 months. For each prediction measure, potential confounders in gender and in‐scanner head motion were regressed out of the measure values.

**TABLE 1:** Descriptions of HCP tasks and performance metrics, where applicable.

Task	Description	Metric(s)
Emotional processing	Matching perceived emotional expression of faces or shapes	Overall accuracy, face accuracy, shape accuracy
Gambling	Simulated rewards and losses based on prediction of number on a card	NA
Language processing	Interleaved questions about a supplied story or mathematical question, provided auditorily	Overall accuracy, story accuracy, math accuracy
Motor	Presented cues invoking directed movement of fingers, toes, or tongue	NA
Relational processing	Differentiating and grouping objects based on texture and shape	Overall accuracy, relational accuracy, match accuracy
Social cognition	Categorizing simulated interaction between objects as random or intentional	Random‐random accuracy, intentional‐intentional accuracy
Working memory	Using recall to determine whether presented pictures match static or dynamic prompts	Overall accuracy, two‐back accuracy, zero‐back accuracy

### 2.7 Prediction Models

Using structural reliance and coupling values from all parcellated regions in each participant, we employed prediction models to compare the effectiveness in predicting cognitive and demographic measures. Linear regression models with least absolute shrinkage and selection operator (LASSO) regularization (due to a large number of features—360 reliance/coupling values) were used for predictions (Tibshirani 1996). Specifically, using regional reliance/coupling values as input features and cognitive/demographic measures as our outcome, prediction models were trained on 80% of the participants, with 20% of the participants left out for testing. Within the training groups, 10‐fold cross‐validation was employed to prevent overfitting and retain generalizability, resulting in an overall Train‐Validation‐Test split of 72%–8%–20%. After training, coefficients were selected from the set that minimized deviance across the cross‐validation folds. The selected model was then applied to testing data, and the performance was measured as the correlation coefficient between the predicted and true performance metrics. This process was repeated 100 times for each set of features, yielding a distribution of prediction performance values from which mean and standard deviation were calculated for comparison between coupling and reliance models. Within each iteration, reliance and coupling were trained and tested on the same train‐test‐validation split, allowing direct comparison of performance across iterations. Using the resulting 100 performance values for both reliance‐ and coupling‐based models, Student's paired *t*‐tests were conducted to compare their performance, with statistical significance set at *p* < 0.05. In cases where all eight fMRI paradigms were used to predict the same measure, multiple comparison correction was employed to define statistical significance at *p* < 0.00625.

## 3 Results

### 3.1 Relative Magnitude Differences Between Regional Reliance and Coupling Values

**FIGURE 2:**
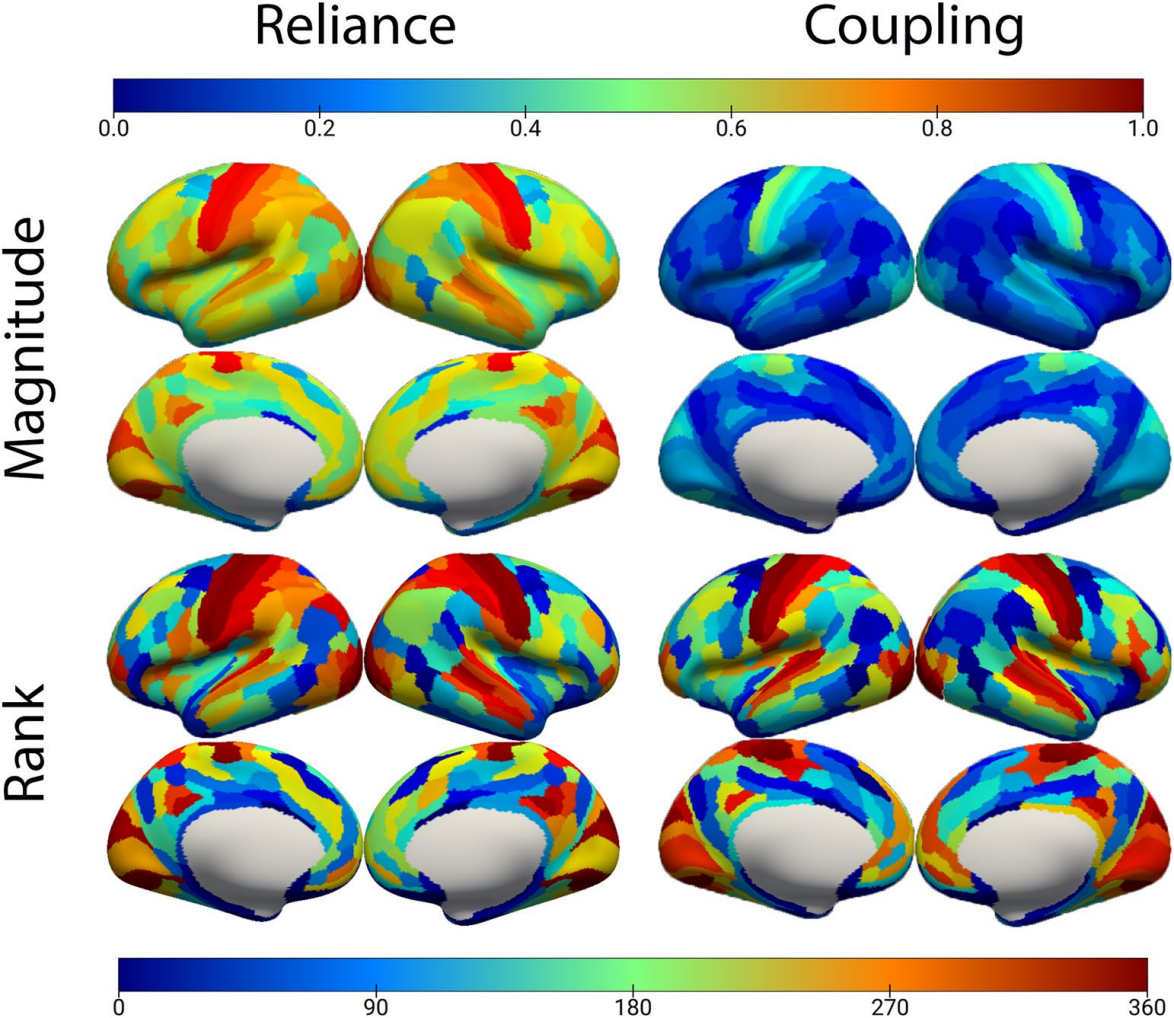
Comparing magnitude patterns of reliance and coupling values. Top left shows the regional average structural reliance values, top right shows the regional average coupling values, bottom left shows the ranked (ascending) structural reliance values, and bottom right shows the ranked (ascending) regional coupling values. For all maps, greater regional coefficient values are displayed as hotter colors.

Structural reliance and coupling values were generated for all parcellated regions and participants using the resting‐state data. Average regional coefficients for both methods were then calculated as the average regional value across all participants, with Figure 2 showing these regional coefficients mapped onto the brain. Group average regional reliance values ranged from 0.14 to 0.90, and group average regional coupling values ranged from 0.06 to 0.53. Participant whole‐brain average values were subsequently generated by calculating the mean across all regional coefficients within each participant. The group mean whole‐brain average reliance value was 0.583 (± 0.0386), and the group mean whole‐brain average coupling value was 0.233 (± 0.0185). Due to the different construction techniques for coupling and reliance coefficients, the magnitudes of regional coefficients are not directly comparable. Thus, Figure 2 also depicts a heat map of regional coefficients ranked by their magnitude within methods, enabling comparison of regional structure–function strength between methods. The maps exhibit some clear differences between the two methods, though unimodal regions tend to exhibit higher values while transmodal regions generally exhibit lower values, as expected (Fotiadis et al. 2024). Regional mean profiles of resting‐state reliance coefficients and coupling coefficients were correlated with a Pearson coefficient of 0.81.

In addition to the resting‐state, mean structural reliance and coupling values were calculated for all seven task paradigms to discern how the structure–function relationship measured by the two techniques changes during different cognitive activities. Again, these analyses serve to demonstrate how the structure–function relationship varies between cognitive tasks and regions, but not between the two methods due to their different construction. Resulting values are shown in Table 2, with regional and ranked heat maps shown in Figures S1 and S2. Furthermore, we calculated the mean standard deviation of participants' whole‐brain reliance/coupling values across different tasks to see how regional reliance and coupling values varied between tasks. Participants' whole‐brain reliance values varied by 0.0325 on average, while participants' whole‐brain coupling values varied by 0.0296 on average. Granting deeper context, participants' regional reliance values varied by 0.2043 across tasks on average, and participants' regional coupling values varied by 0.0834, indicating high variation in the structure–function relationship at the regional level between different cognitive tasks and demands.

**TABLE 2:** Magnitude of mean coupling and reliance values across different HCP task paradigms.

Task paradigm	Mean regional reliance coefficient	Mean regional coupling coefficient
Rest	0.583 (0.21)	0.233 (0.12)
Emotion	0.542 (0.30)	0.150 (0.11)
Gambling	0.558 (0.29)	0.162 (0.11)
Language	0.568 (0.28)	0.177 (0.11)
Motor	0.582 (0.26)	0.193 (0.11)
Relational	0.574 (0.29)	0.159 (0.10)
Social	0.600 (0.27)	0.179 (0.10)
Working memory	0.585 (0.26)	0.192 (0.11)

*Note:* Parenthesized values are the standard deviation of regional reliance/coupling values within participants.

### 3.2 Structural Reliance Outperforms Coupling in Predicting fMRI Task Performance

**FIGURE 3:**
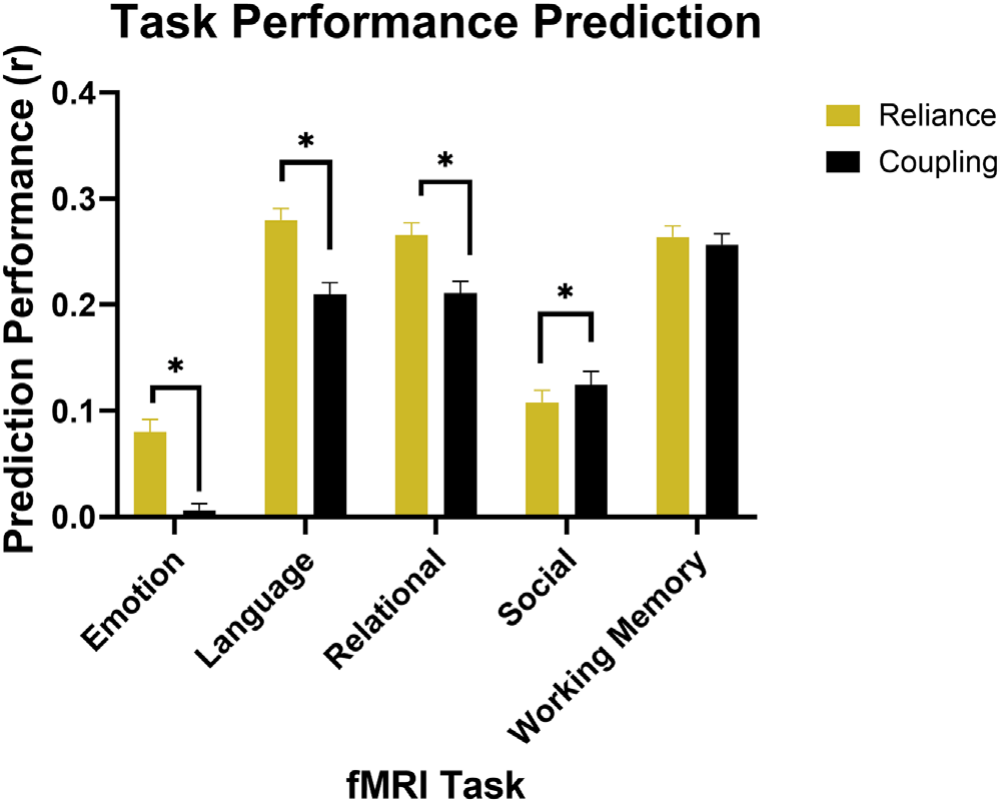
Performance of reliance and coupling techniques in predicting fMRI task accuracy. Model performance was computed as the correlation between true fMRI task accuracy and predicted accuracy from LASSO‐regularized model fits to either reliance or coupling regional coefficients. The average correlation value was then calculated for the overall performance value, shown by box height. The 95% confidence intervals for prediction performances are shown as well. *Significant difference in performance at *p* < 0.05.

We aimed to determine which method more accurately predicted performance on tasks conducted during fMRI data acquisition. Prediction performance for reliance and coupling models across different fMRI task paradigms is shown in Figure 3 and Table S2.

Structural reliance proved significantly more effective in predicting task performance in three of the five task paradigms, including emotional processing, language, and relational processing. Notably, the social cognition task, which was the only task in which standard coupling significantly outperformed reliance, was the only task used for prediction without an overall accuracy metric. Instead, overall performance for this task was calculated as the sum of correct classifications across the two types of prompts subtracted by the number of incorrect classifications. The remaining task paradigm, working memory, saw no significant difference in prediction performance between reliance and standard coupling.

### 3.3 Structural Reliance Predicts General Cognitive Ability Better Than Coupling

**FIGURE 4:**
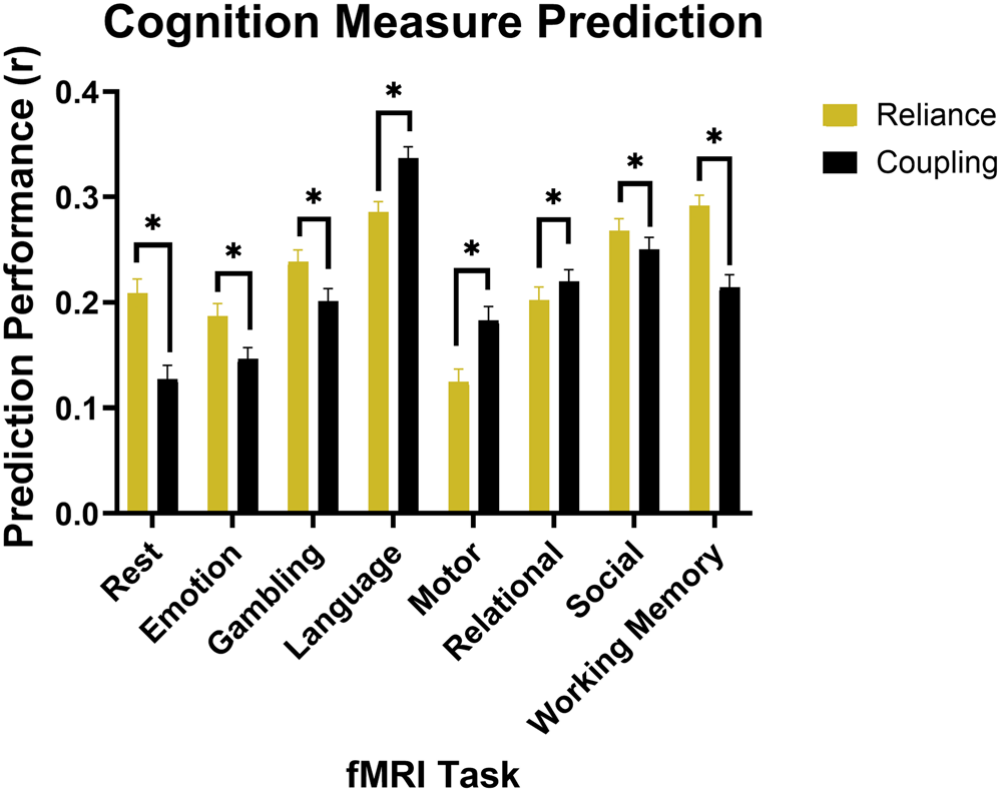
Performance of reliance and coupling techniques in predicting cognition outcomes. Performance was computed as the correlation between the true cognition measure defined as NIH Toolbox Cognition Total Composite Score and the predicted cognition value from LASSO‐regularized generalized model fits to either reliance or coupling regional coefficients. The average correlation value was then calculated for the overall performance value, shown by box height. The 95% confidence intervals for prediction performances are shown as well. *Significant difference in performance at *p* < 0.00625.

Performances of structural reliance and SC‐FC coupling values in predicting NIH Toolbox Cognition Total Composite Score across different tasks are shown in Figure 4 and Table S3. Performance was assessed by computing the correlation between the predicted and true cognition values.

Structural reliance performed significantly better than correlation‐based coupling across the resting‐state data and four of the seven task data including emotional processing, gambling, social cognition, and working memory. Coupling was significantly more effective at predicting cognition during the language, motor, and relational processing tasks. When comparing performance across tasks, the resting‐state paradigm was least effective in predicting cognition, while tasks like language, social cognition, and working memory were most effective.

### 3.4 Structural Reliance Outperforms Coupling in Predicting Demographic Measures

**FIGURE 5:**
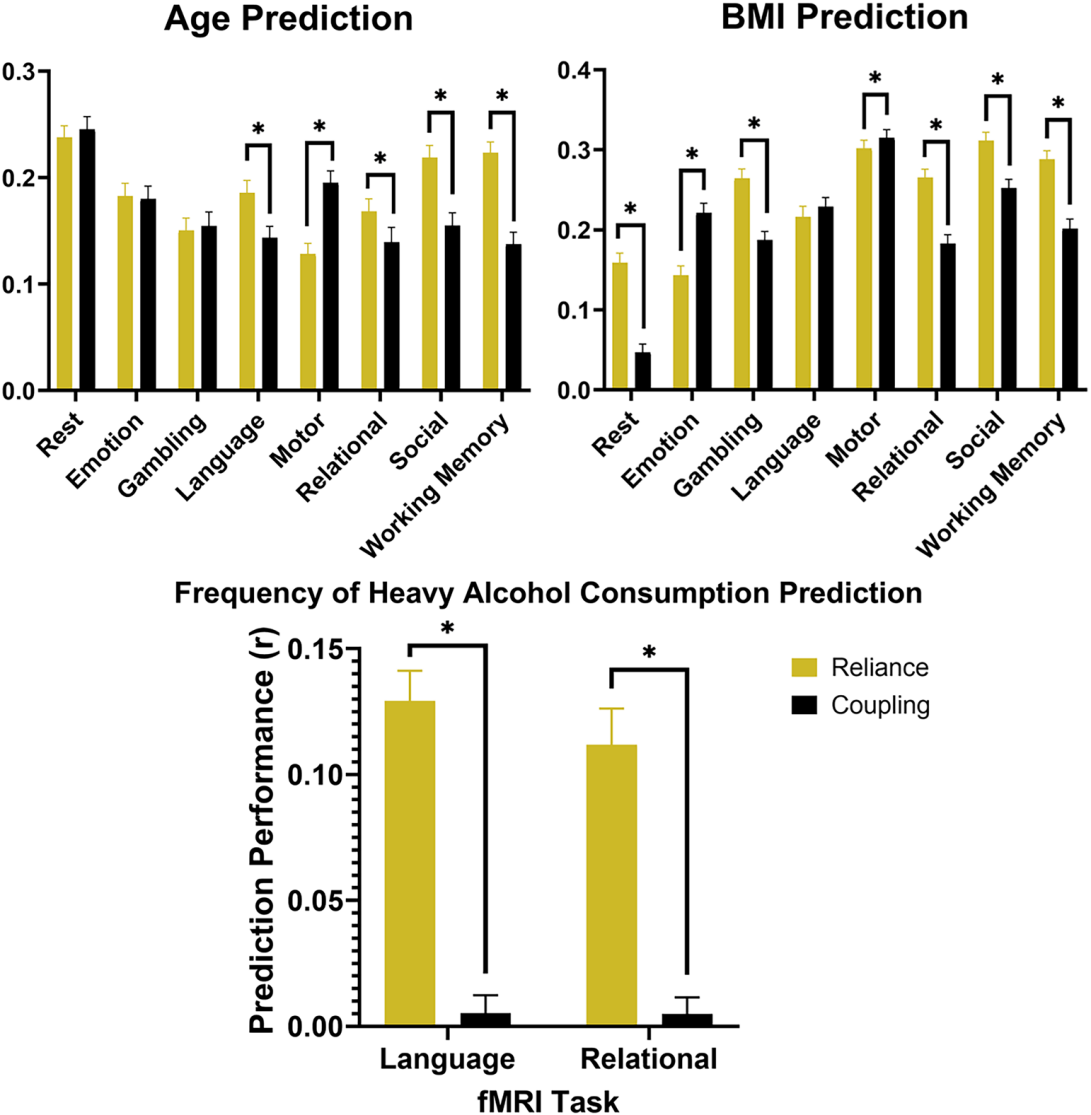
Performance of reliance and coupling techniques in predicting participant age, body mass index (BMI), and frequency of heavy alcohol consumption. Performance was computed as the correlation between true measures and predicted values from LASSO‐regularized model fits to either reliance or coupling regional coefficients. The average correlation value was then calculated for the overall performance value, shown by box height. The 95% confidence intervals for prediction performances are shown as well. *Significant difference in performance at *p* < 0.00625.

Prediction results for age, BMI, and alcohol consumption are shown in Figure 5 and Table S4.

As shown, structural reliance was significantly more effective in predicting participant age across four task paradigms including language, relational processing, social cognition, and working memory. Correlation‐based coupling, on the other hand, significantly outperformed reliance in just the motor task. Resting‐state, emotional processing, and gambling tasks saw statistically similar performance between the two techniques. Resting‐state was the most effective paradigm for predicting age, with the other tasks exhibiting similar predictive power to each other that was slightly decreased compared to the resting‐state data.

Similarly, in predicting BMI, structural reliance proved to be significantly more effective across four task paradigms including gambling, relational processing, social cognition, and working memory, as well as resting‐state data. Coupling statistically outperformed reliance during emotional processing and motor tasks, and there was no significant difference in performance during the language task. Regarding effectiveness of each task in predicting BMI, resting‐state provided the lowest predictive performance, whereas motor and social cognition tasks proved to be the most effective.

Compared to previous measures, frequency of heavy alcohol consumption was more difficult to predict using reliance or coupling measures across most tasks. Neither reliance or coupling coefficients generated during resting‐state, emotional processing, gambling, motor, social cognition, and working memory tasks exhibited performance values greater than 0.1. Among the remaining paradigms, structural reliance was significantly more effective at predicting frequency of heavy alcohol consumption during language and relational processing tasks. See precise prediction performance values for each paradigm in Table S4.

## 4 Discussion

Despite numerous studies examining the relationship between MRI‐measured SC and FC, both the nature of this relationship and its relevance to brain function are not fully understood (Kong et al. 2021; Liu et al. 2022; Popp et al. 2024; Wang et al. 2023). Here, we explored this relationship through the introduction of a new interpretable method and evaluation of its utility across different fMRI paradigms. This method, called structural reliance, exhibited different magnitude patterns across the cortex and generally outperformed standard coupling in predicting a variety of behavioral variables representing various cognitive and demographic measures. Importantly, this general performance increase was observed after global signal regression, ensuring that performance is reliant on the structure–function relationship rather than global signal. Without SC values as weights, the generated structural reliance values would be zero as a reflection of the null global signal.

Though construction of the regional coefficients between reliance and coupling is different, we were able to compare relative magnitudes of regional structure–function correspondence between reliance and coupling. The two methods resulted in notably different patterns across the brain, while some expected similarities persisted as increased magnitudes in unimodal regions and decreased coefficient values in transmodal regions (Fotiadis et al. 2024). These differing patterns serve to demonstrate that coupling and reliance extract unique information in their quantification of the structure–function relationship. One key difference is the similar magnitude of mean reliance values across all fMRI paradigms, while the mean coupling values during fMRI tasks are notably lower than at rest. During different brain states or functional patterns, decreased mean coupling values could indicate a decreased utilization of the structural connectome. Mean reliance values are more consistent across the different states, potentially indicating a more consistent overall reliance on the structural connectome, even if different connections are active/inactive.

We also compared whole‐brain average and regional coefficients across task paradigms, finding consistent whole‐brain average values between tasks despite highly variable regional coefficients between fMRI paradigms and different cognitive demands (Griffa et al. 2022; Liu et al. 2022). Given the dynamic, flexible functional patterns evoked by varying cognitive demands, it is expected that different cognitive demands would result in functional patterns that adhere to the structural connectome differently (Gupta et al. 2022; Khodaei et al. 2023; Liu et al. 2022; McIntyre et al. 2024; Shappell et al. 2021). This phenomenon is further exemplified by the regional heatmaps of regional coefficients and coefficient magnitude ranks, which exhibited notable differences both between coupling and reliance and between different fMRI paradigms (Figures S1 and S2).

Examining how reliance and coupling values are related to behavior was also critical to establish structural reliance as a viable tool for quantifying the structure–function relationship. We investigated three categories of behavioral variables: fMRI task performance, overall cognition, and demographic measures. Based on the predictions by linear models trained on reliance and coupling values, structural reliance was significantly more effective in three of five paradigms in predicting task accuracy, five of eight paradigms in predicting overall cognition, four of eight paradigms in predicting age, five of eight paradigms in predicting BMI, and two of two viable paradigms in predicting frequency of heavy alcohol consumption. Meanwhile, SC‐FC coupling was more effective in one of five, three of eight, one of eight, two of eight, and zero of two paradigms, respectively. Combined, structural reliance significantly outperformed coupling in 19 of 31 models, while coupling outperformed reliance in only seven of 31 models. Importantly, the two techniques performed statistically similarly in only five of the models, further indicating that structural reliance and correlation‐based coupling extract unique information about the structure–function relationship.

Reliance values generally outperformed SC‐FC coupling in predicting behavioral outcomes. Given the distinct patterns of structure–function patterns obtained by reliance, this could again suggest that structural reliance may serve as a viable alternative to quantify the structure–function relationship. We contend that structural reliance embraces the dynamics of brain communication and limits both false positive (low SC, high FC) and false negative (high SC, low FC) coupling values by considering a weighted sum of all regional activity during calculations. False positives, which may present due to unconnected regions exhibiting similar activity resulting from communication with a mutual neighbor, are mitigated during time series reconstruction by small SC values as weights. False negatives, which may result from connections between different functional networks, are reduced through the influence of multiple other connections with functionally homogeneous regions.

Finally, through our comparison we also demonstrated the impact of different fMRI task paradigms on structure–function patterns in the brain and how they relate to behavior. In predicting overall cognition, both structural reliance and SC‐FC coupling were increasingly effective during language, working memory, and social cognition tasks. The increased cognitive loads during these task paradigms likely evoke structure–function patterns more reflective of overall cognitive ability (Barch et al. 2013). Age was best predicted during rest, perhaps indicating a more general, abstract measure not easily represented during specific cognitive tasks. The motor task, alternatively, was the most effective paradigm for predicting BMI, as could be anticipated given the association between physical activity and BMI (Grasdalsmoen et al. 2019). Finally, the two paradigms which proved effective in predicting frequency of healthy alcohol consumption were language and relational processing. The difficulty in predicting alcohol consumption can likely be attributed to the complex nature and heavy variety in factors contributing to alcohol consumption (Chartier et al. 2017; Perry and Carroll 2008). Overall, the variation in predictive performance across the fMRI paradigms speaks to the complex nature of functional brain activity and its adherence to structure, further demonstrating the importance of cognitive demands when investigating the structure–function relationship. Of note, though each fMRI paradigm had a different duration, we did not identify any overarching patterns of predictive performance based on duration. Instead, the effectiveness of each paradigm in predicting behavior appears related to the connection between behavior and fMRI task.

### 4.1 Future Directions and Alternative Applications

Structural reliance was introduced here as a complementary or alternative technique to current methods, aiming to enhance our understanding of how structural connections in the brain shape functional activity. As such, we compared reliance to correlation‐based coupling that utilized direct SC values. Often, structure–function studies have employed alternative methods like hierarchical/spatial coupling patterns or graph theory measures to examine how the relationship is connected to behavior (Popp et al. 2024; Rubinov and Sporns 2010; Seguin et al. 2020; Suarez et al. 2020; Wang et al. 2023; Zamani Esfahlani et al. 2022). Such techniques are valuable in understanding the flow and accumulation of information in networks like the brain. For instance, path length helps to account for functional similarity resulting from communication via mutual neighbors. Here, however, structural reliance was introduced and tested as a technique to measure the relationship between structural and functional connections in the brain. Consequently, these techniques were not incorporated into this analysis, as the focus centered around how structural reliance compares to correlation‐based coupling in describing functional activity resulting from direct structural connections. In doing so, we demonstrate the utility of structural reliance and potentiate its use in many current benchmark techniques. Given the improved predictive performance of structural reliance compared to standard correlation‐based coupling, it may provide benefits over standard correlation‐based coupling in many benchmark methods. Graph theory metrics approaches could use distance/similarity metrics as weights in place of SC values within the reliance framework, and cortical/spatial organization analysis techniques could utilize reliance coefficients in place of standard coupling coefficients (Baum et al. 2020; Popp et al. 2024; Vazquez‐Rodriguez et al. 2019). Future work should investigate the utility of structural reliance alongside such established techniques or other unique coupling strategies to evaluate the structure–function relationship and its implications on behavior.

In addition to its potential as an alternative to standard correlation‐based coupling, the framework of structural reliance allows for more intriguing explorations limited by correlation‐based coupling. The construction of the predicted time series allows for more flexible analysis of structural connections between regions. By exploring different weighting techniques, one could identify particular patterns of structural adherence, inactive and inhibitory connections, and active subnetworks of the structural connectome. Moreover, by identifying portions of the BOLD series where the predicted and true time series diverge, one can explore how these patterns and connections evolve over time without worrying about limitations like window size selection. Though some previous research has explored standard coupling across time, these techniques are limited to evaluating the strength of the relationship over time (Liu et al. 2022; Zhang et al. 2024). Again, the framework of structural reliance could instead enable feature selection or weighting alternatives that reveal specific connection dynamics.

### 4.2 Limitations

Though its strong performance in predicting behavior is encouraging, there are a few important considerations regarding the effectiveness of structural reliance. By constructing the predicted BOLD series using derivatives at each TR, the introduction of noise and instability becomes a concern. This may be mitigated by the nature of summing values across the multitude of regions; however, longer window approaches were ineffective in improving performance (Table S5). Additionally, structural connections are highly complex, and the linear weighting in structural reliance may not be ideal for handling regional SC sparsity differences or nonlinear relationships between structure and communication. Future work should investigate potential density controls and stabilizers to account for nonlinear patterns and SC distribution differences between regions.

## 5 Conclusion

In this study, we presented a novel technique for quantifying the structure–function relationship in the human brain called structural reliance. Through comparison of relative magnitude values, we demonstrated that structural reliance and coupling reveal different patterns of structure–function correspondence in the brain. Additionally, linear models were used to predict various cognitive tasks as well as some extensively used behavioral outcomes from reliance and coupling values. Structural reliance significantly outperformed coupling in 19 of the 31 prediction models, whereas coupling outperformed reliance in only seven prediction models, with the remaining models yielding similar performances. This stark difference in performance indicates that structural reliance may offer a more appropriate measure of the structure–function relationship, effectively capturing cognitively and behaviorally relevant information that eludes traditional coupling methods. Alongside our comparison of the two structure–function techniques, we also assessed the effectiveness of various task paradigms in predicting various cognitive tasks and demographic measures. The variability in model performance across different tasks highlights the importance of considering the functional patterns elicited by each task when investigating structure–function associations and their relation to behavioral outcomes.

## Funding

This work was supported by the National Institutes of Health (P50AA026117, K25‐EB032903‐01, K25AG090707) and National Science Foundation (GRFP 2024377885).

## Disclosure

I confirm that this stipulation is not applicable, as my article reports no human subjects research. All data is from a publicly available dataset (Human Connectome Project).

## Conflicts of Interest

The authors declare no conflicts of interest.

## Supporting information


**Table S1:** fMRI time series and sample size information for each task.
**Table S2:** Summary of predictive performance for coupling and reliance across different fMRI tasks, shown as mean (standard deviation). Performance was tracked as the correlation between true fMRI task accuracy and predicted accuracy from LASSO‐regularized generalized linear model fit on either reliance or coupling regional coefficients. *Significantly greater performance in task accuracy prediction determined by a two‐tailed student *t*‐test with *p* < 0.05.
**Table S3:** Summary of predictive performance for coupling and reliance across different fMRI tasks, shown as mean (standard deviation). Performance was tracked as the correlation between true NIH Toolbox Cognition Total Composite scores and predicted values from LASSO‐regularized generalized linear model fit on either reliance or coupling regional coefficients. *Significantly greater performance in cognition prediction determined by a two‐tailed student *t*‐test with *p* < 0.05.
**Table S4:** Summary of predictive performance for coupling and reliance across different fMRI tasks, shown as mean (standard deviation). Performance was tracked as the correlation between various lifestyle measure values and predicted values from LASSO‐regularized generalized linear model fit on either reliance or coupling regional coefficients. The various demographic measures were age in years, body mass index (BMI), and total drinks consumed in the last 7 days. *Significantly greater performance in lifestyle measure prediction determined by a two‐tailed student *t*‐test with *p* < 0.05.
**Table S5:** Investigation of longer‐window approaches for structural reliance time series construction. We evaluated windows of different lengths of TR, calculating the BOLD derivative as the difference in BOLD divided by the duration of the window. Both mean reliance values and working memory task accuracy prediction values are shown for different window lengths.
**Figure S1:** Heatmaps of mean reliance (left) and coupling (right) coefficients for each fMRI paradigm. Hotter colors indicate greater values, while colder colors indicate lower values. Reliance and coupling maps were calculated as the mean regional coefficients across all participants.
**Figure S2:** Heatmaps of relative reliance (left) and coupling (right) coefficients for each fMRI paradigm. Hotter colors indicate greater values, while colder colors indicate lower values. Reliance and coupling maps were calculated as the mean regional coefficients across all participants, after which mean values were sorted and assigned ascending rank values where rank 360 indicates the highest mean regional coefficient.

## Data Availability

The data that support the findings of this study are available from the corresponding author upon reasonable request.
