# Supplementary material for: Introducing Structural Reliance: A New Method to Assess Structure–Function Coupling in the Brain
Source: Hum Brain Mapp. 2026 Mar 26;47(5):e70499. doi: 10.1002/hbm.70499 (PMC13081694; doi:10.1002/hbm.70499)
Supplement: Supplementary file 1 — Table S1: fMRI time series and sample size information for each task. Table S2: Summary of predictive performance for coupling and reliance across different fMRI tasks, shown as mean (standard deviation). Performance was tracked as the correlation between true fMRI task accuracy and predicted accuracy from LASSO‐regularized generalized linear model fit on either reliance or coupling regional coefficients. *Significantly greater performance in task accuracy prediction determined by a two‐tailed student t‐test with p < 0.05. Table S3: Summary of predictive performance for coupling and reliance across different fMRI tasks, shown as mean (standard deviation). Performance was tracked as the correlation between true NIH Toolbox Cognition Total Composite scores and predicted values from LASSO‐regularized generalized linear model fit on either reliance or coupling regional coefficients. *Significantly greater performance in cognition prediction determined by a two‐tailed student t‐test with p < 0.05. Table S4: Summary of predictive performance for coupling and reliance across different fMRI tasks, shown as mean (standard deviation). Performance was tracked as the correlation between various lifestyle measure values and predicted values from LASSO‐regularized generalized linear model fit on either reliance or coupling regional coefficients. The various demographic measures were age in years, body mass index (BMI), and total drinks consumed in the last 7 days. *Significantly greater performance in lifestyle measure prediction determined by a two‐tailed student t‐test with p < 0.05. Table S5: Investigation of longer‐window approaches for structural reliance time series construction. We evaluated windows of different lengths of TR, calculating the BOLD derivative as the difference in BOLD divided by the duration of the window. Both mean reliance values and working memory task accuracy prediction values are shown for different window lengths. Figure S1: Heatmaps of mean reliance [file HBM-47-e70499-s001.docx]

**Supplementary**

**Further detail on structural connectivity calculation.**

We used the MMP atlas, which parcellates the brain based on both structural and functional connections, enabling a more reliable assessment of structure-function coupling (Glasser et al., 2016). More specifically, probabilistic tractography was run using Probtrackx2 with loop checking, and curvature and fiber thresholds of 0.2 and 0.01, respectively. Streamlines were generated with a step length of 0.5 mm and up to 2000 steps per streamline. Seed mask vertices were sampled at 2 mm resolution, and the initial step was forced at the seed location. Connectivity from each seed vertex to all other vertices in the target space was stored using omatrix1 to generate the dense connectome for each individual.

The traditional coupling approach used for comparison herein was conducted via the Pearson correlation. In testing the Spearman correlation in fMRI task prediction, the absolute magnitude of coupling coefficients was decreased compared to Pearson-based coefficients (Emotion: 0.109, Language: 0.121, Relational: 0.116, Social: 0.124, Working Memory: 0.133). Prediction performance of the Spearman-based coefficients was generally statistically similar or decreased compared to Pearson-based coefficients (Emotion: 0.00009, Language: 0.217, Relational: 0.173, Social: 0.142, Working Memory: 0.154).

**Table S1.** fMRI time series and sample size information for each task.

| Task Paradigm | Number of Time Points | Number of Participants |
| --- | --- | --- |
| Resting-State | 1200 | 1019 |
| Emotional Processing | 176 | 1009 |
| Gambling | 253 | 1026 |
| Language | 316 | 1015 |
| Motor | 284 | 1025 |
| Relational Processing | 232 | 1005 |
| Social Cognition | 274 | 1013 |
| Working Memory | 405 | 1022 |

**Table S2**. Summary of predictive performance for coupling and reliance across different fMRI tasks, shown as mean (standard deviation). Performance was tracked as the correlation between true fMRI task accuracy and predicted accuracy from LASSO-regularized generalized linear model fit on either reliance or coupling regional coefficients. * Indicates significantly greater performance in task accuracy prediction determined by a two-tailed student t-test with p < 0.05.

| fMRI Task | Reliance Performance | Coupling Performance |
| --- | --- | --- |
| Emotion | 0.080 (0.06)* | 0.006 (0.03) |
| Language | 0.280 (0.06)* | 0.210 (0.05) |
| Relational | 0.266 (0.06)* | 0.211 (0.06) |
| Social | 0.108 (0.06) | 0.125 (0.06)* |
| Working Memory | 0.264 (0.05) | 0.256 (0.05) |

**Table S3**. Summary of predictive performance for coupling and reliance across different fMRI tasks, shown as mean (standard deviation). Performance was tracked as the correlation between true NIH Toolbox Cognition Total Composite scores and predicted values from LASSO-regularized generalized linear model fit on either reliance or coupling regional coefficients. * Indicates significantly greater performance in cognition prediction determined by a two-tailed student t-test with p < 0.05.

| fMRI Task | Reliance | Coupling |
| --- | --- | --- |
| Rest | 0.209 (0.07)* | 0.127 (0.07) |
| Emotion | 0.187 (0.06)* | 0.146 (0.05) |
| Gambling | 0.238 (0.06)* | 0.201 (0.06) |
| Language | 0.286 (0.05) | 0.337 (0.05)* |
| Motor | 0.125 (0.06) | 0.183 (0.07)* |
| Relational | 0.202 (0.06) | 0.220 (0.06)* |
| Social | 0.268 (0.06)* | 0.250 (0.06) |
| Working Memory | 0.292 (0.05)* | 0.214 (0.06) |

**Table S4**. Summary of predictive performance for coupling and reliance across different fMRI tasks, shown as mean (standard deviation). Performance was tracked as the correlation between various lifestyle measure values and predicted values from LASSO-regularized generalized linear model fit on either reliance or coupling regional coefficients. The various demographic measures were age in years, body mass index (BMI), and total drinks consumed in the last seven days. * Indicates significantly greater performance in lifestyle measure prediction determined by a two-tailed student t-test with p < 0.05.

| Output Measure | Age | | BMI | | Alcohol Consumption | |
| --- | --- | --- | --- | --- | --- | --- |
| fMRI Task | Reliance | Coupling | Reliance | Coupling | Reliance | Coupling |
| Rest | 0.238 (0.05) | 0.245 (0.06) | 0.160 (0.06)* | 0.047 (0.05) | 0.043 (0.05)* | 0.023 (0.04) |
| Emotion | 0.183 (0.06) | 0.180 (0.06) | 0.143 (0.06) | 0.221 (0.06)* | 0.073  (0.06)* | 0.047  (0.06) |
| Gambling | 0.150 (0.06) | 0.155 (0.07) | 0.264 (0.06)* | 0.187 (0.05) | 0.028 (0.05) | 0.044 (0.05) |
| Language | 0.186 (0.06)* | 0.144 (0.05) | 0.216 (0.07) | 0.229 (0.06) | 0.129  (0.06)* | 0.001  (0.04) |
| Motor | 0.128 (0.05) | 0.195 (0.06)* | 0.302 (0.05) | 0.315 (0.05)* | 0.033 (0.05) | 0.043 (0.06) |
| Relational | 0.168 (0.06)* | 0.139 (0.07) | 0.265 (0.05)* | 0.183 (0.06) | 0.112  (0.07)* | 0.005  (0.03) |
| Social | 0.219 (0.06)* | 0.155 (0.06) | 0.311 (0.05)* | 0.252 (0.06) | 0.063  (0.05) | 0.072  (0.06) |
| Working Memory | 0.223 (0.05)* | 0.137 (0.06) | 0.288 (0.06)* | 0.202 (0.06) | 0.003  (0.04) | 0.041  (0.05)* |

**Table S5**. Investigation of longer-window approaches for structural reliance time series construction. We evaluated windows of different lengths of TR, calculating the BOLD derivative as the difference in BOLD divided by the duration of the window. Both mean reliance values and working memory task accuracy prediction values are shown for different window lengths.

| Window Length (TR) | Mean Reliance Value | Mean Predictive Performance |
| --- | --- | --- |
| 1 | 0.585 (0.0393) | 0.264 (0.05) |
| 5 | 0.582 (0.0391) | 0.260 (0.05) |
| 10 | 0.574 (0.0385) | 0.258 (0.05) |
| 25 | 0.495 (0.0370) | 0.256 (0.05) |

**
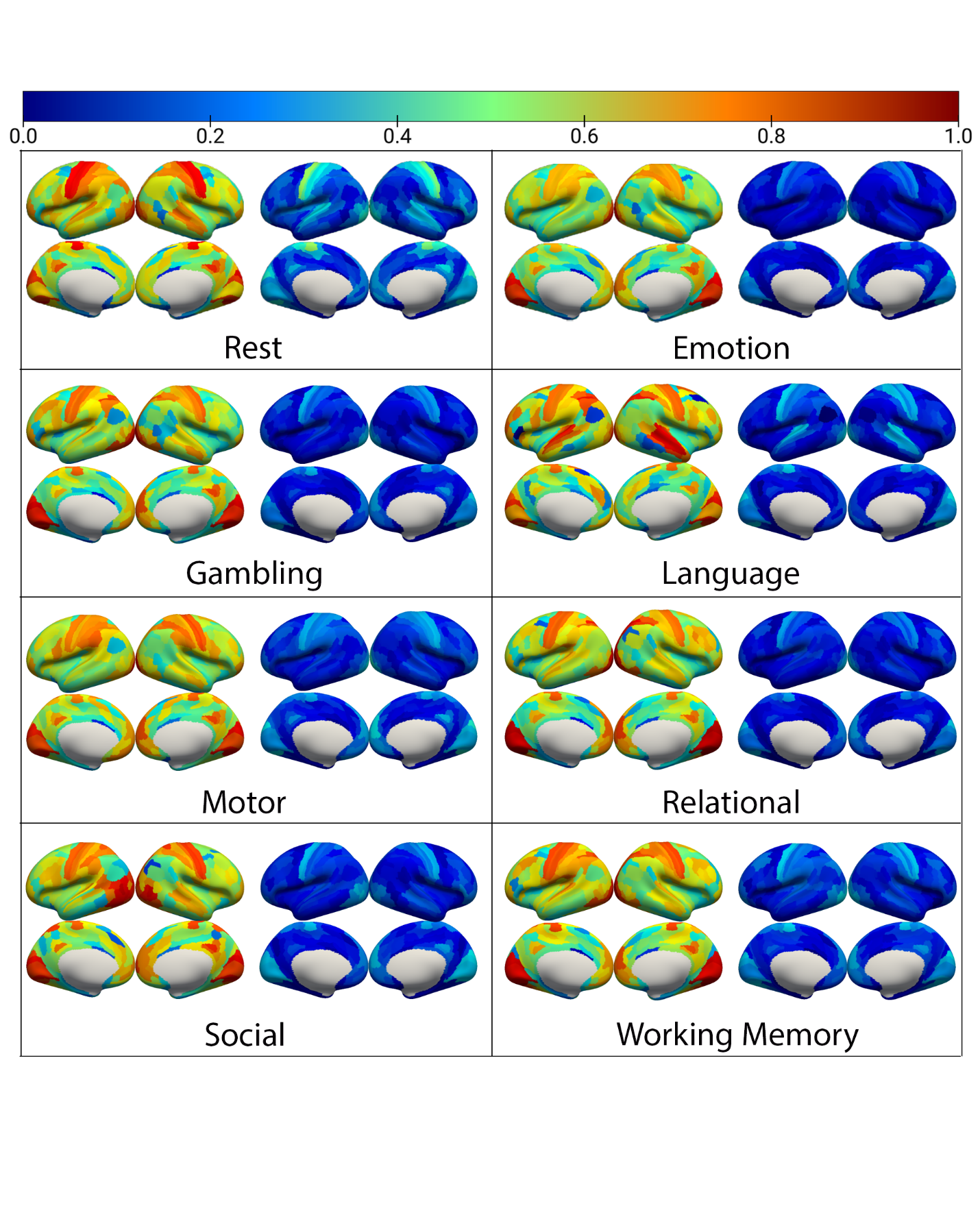
Figure S1.** Heatmaps of mean reliance (left) and coupling (right) coefficients for each fMRI paradigm. Hotter colors indicate greater values, while colder colors indicate lower values. Reliance and coupling maps were calculated as the mean regional coefficients across all participants.

**
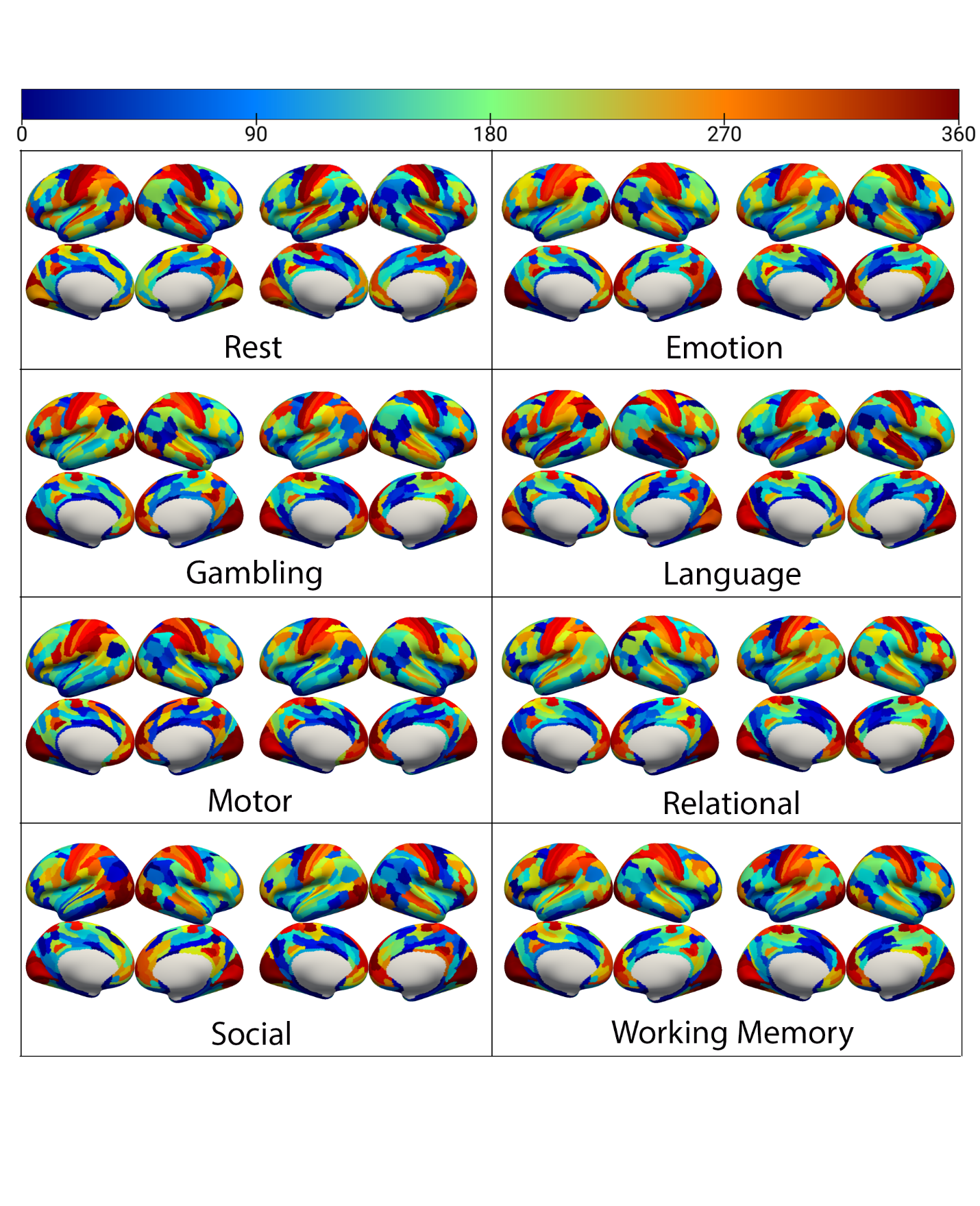
Figure S2.** Heatmaps of relative reliance (left) and coupling (right) coefficients for each fMRI paradigm. Hotter colors indicate greater values, while colder colors indicate lower values. Reliance and coupling maps were calculated as the mean regional coefficients across all participants, after which mean values were sorted and assigned ascending rank values where rank 360 indicates the highest mean regional coefficient.
